# The State Board of Examiners

**Published:** 1872-04

**Authors:** 


### The State Board of Examiners

Will hold the semi-annual meeting on Wednesday, June 5th, at 10 o'clock A. M., in the Dental College in this city.

Let all who desire to meet the Board notify some member of it some time previous to the meeting if possible. We trust that every honorable member of the profession in the State will appreciate the very great influence for good the law regulating the practice of dentistry is exercising. The dental profession in Ohio is now occupying an enviable position, and is rising higher and higher every day, and very largely is this attributable to this law. Members all over the State are reaching for higher attainments and pushing their way upward. The multiplication and introduction of quacks and professional imbeciles has been quite effectually checked, and thus thousands in our State are comparatively free from mutilations and maltreatment that were a few years ago perpetrated upon them without let or hindrance. The people in Ohio are to-day appreciating and valuing their teeth more than ever before, and are more anxiously inquiring what they shall do to save them, and are daily demanding better practice, and this has its influence upon the profession, and is a stimulus that can not be disregarded. He who arrays himself against good and wholesome regulations is an enemy to the people and to his profession.

### The Ohio Dental College.

At the close of the last session of this institution the Class of Chemistry was made vacant by the resignation of Prof. D. Vaughn, who so ably filled the position during the last year; and that of Mechanical Dentistry, by the resignation of Prof. C. M. Wright, made necessary as will be seen by reference to another editorial in this number of the Register.

The chair of chemistry has been filled by the election of Dr. J. S. Cassidy, a graduate of this institution, who has made chemical science a study for several years, and will doubtless fill the position with credit to himself and great benefit to his pupils. The chair of Mechanical Dentistry was filled by the appointment of Dr. Corydon Palmer, than whom in practical dentistry there is no more thorough and perfect teacher in our Country, or indeed in any other country. We are fully conscious that these appointments will bring much strength and prestige to the college, and we know that the profession will with very great gratification receive knowledge of the fact. They will both take part in the spring course.

### Hymenial.

Married on the 28th of April, Dr N. W. Williams and Miss Belle Smith, of Xenia, Ohio, at the residence of the bride's father.

We extend to the Doctor and his fair bride our most hearty congratulations, and the strongest wish for that unbounded bliss for which they are booked. And in their departure for a foreign land they will bear with them the kindest regards and best wishes of the entire profession of the West.

Prof. W. H. Atkinson will be present at the spring term in the college, on the 6th of May, and continue for a short time, to give instruction to the class, in practice and principles. His methods, as are generally known in the profession, are very practical and thorough.

